# Supplementary material for: Mantle exhumation at magma-poor rifted margins controlled by frictional shear zones
Source: Nat Commun. 2022 Mar 28;13:1634. doi: 10.1038/s41467-022-29058-1 (PMC8960832; doi:10.1038/s41467-022-29058-1)
Supplement: Supplementary file 1 — Supplementary material [file 41467_2022_29058_MOESM1_ESM.pdf]

Supplementary Materials for

Mantle exhumation at magma-poor rifted margins controlled by frictional shear zones

Thomas Theunissen*, Ritske S. Huismans

*Corresponding author. Email: thomas.theunissen@uib.no

This PDF file includes:

Figs. S1 to S15
Tables S1 to S3
Supplementary References

Other Supplementary Materials for this manuscript include the following:

Movies S1 to S2
Movies of the set of models exploring the sensitivity to strain weakening, full extension rate and thermal conductivity (Fig. S9 to S15) (On figshare.com).

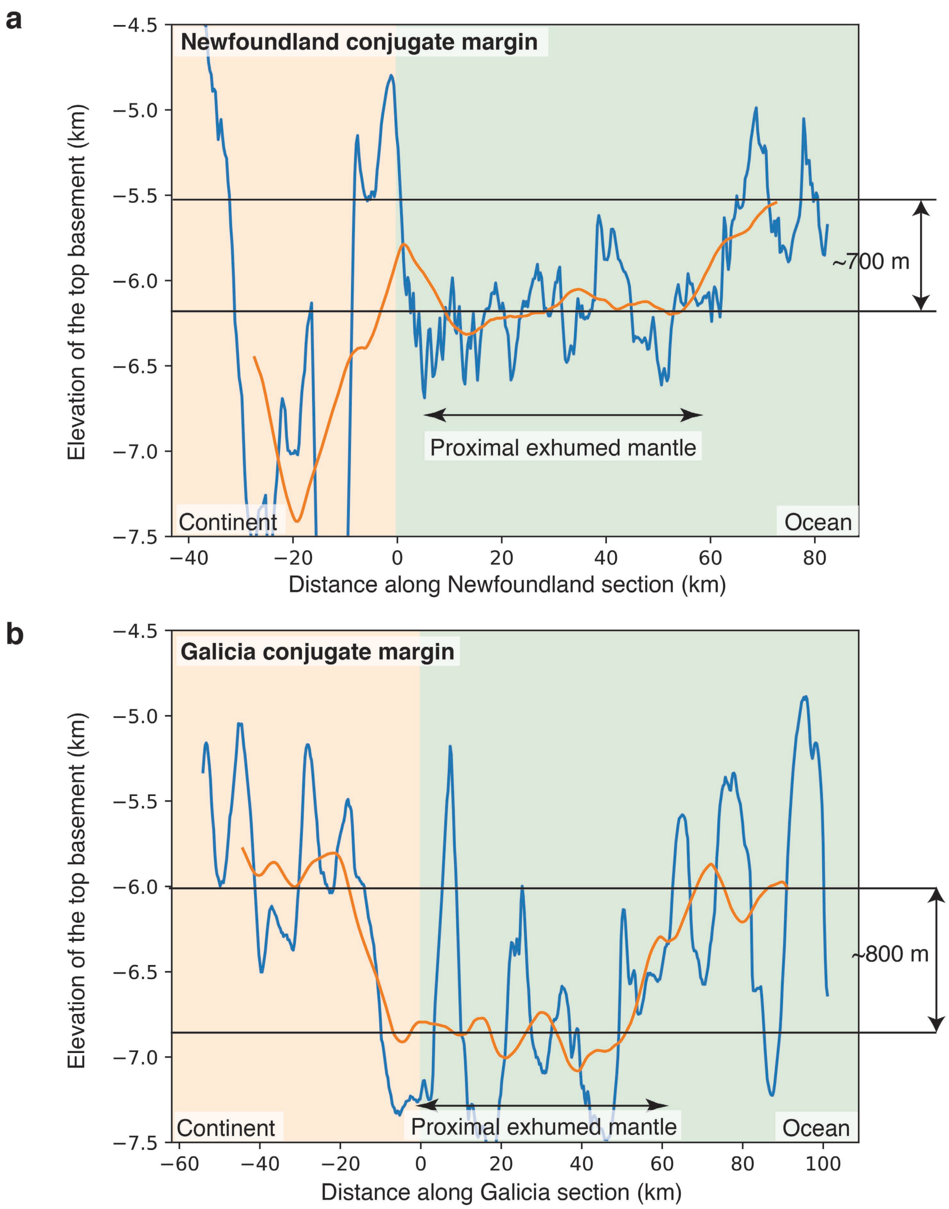

Fig. S1.

Analysis of the top basement elevation of the transitional domain of the Galicia-Newfoundland conjugate margins. a, Newfoundland; **b,** Galicia. The rifted continental crust is on the left side (distances < 0) while the exhumed mantle domain is on the right side (distances > 0). The orange curve represents the average elevation calculated from the observed elevation of the top basement (blue curve, similar in Fig. 2c). Black horizontal lines highlight the differences in elevation between proximal and distal part of the transition zone. This difference is close to $h_c(\rho_{oc} - \rho_M)/(\rho_W - \rho_M)$ = 840 m where hc is an oceanic crustal thickness of 4 km, ρ_{oc} is the average density of the oceanic crust (2900 kg/m^3), ρ_M is a characteristic mantle density for the compensated lithospheric column (3400 kg/m^3), and ρ_W the water density (1030 kg/m^3). Note that the difference in elevation between the two margins is the consequence of positive dynamic topography beneath Newfoundland and negative beneath Galicia most likely caused by the presence of deep mantle heterogeneities[1].

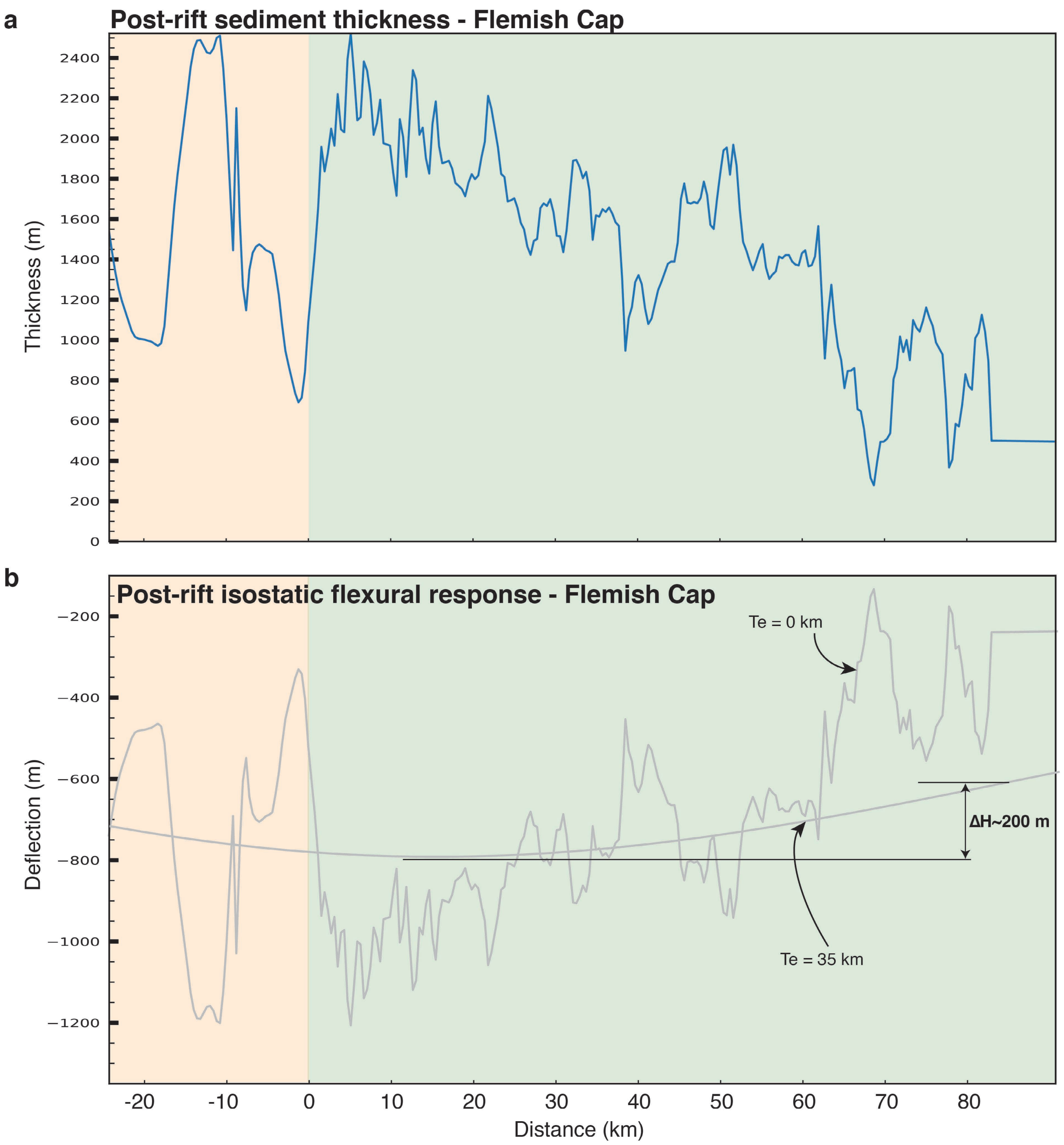

Fig. S2.

Isostatic flexural response to post-rift sediment loading along the Flemish Cap transitional exhumed mantle domain. a, Post-rift sediment thickness; **b,** Post-rift isostatic flexural response. Because the proximity with the continental lithosphere we select 35 km thick elastic thickness that slightly overestimates the result of the following relationship $Te = (2.7 \pm 0.15)\sqrt{\Delta t}$ (Δt in Ma, here 121 Ma = age of crustal breakup) that is defined for oceanic lithosphere far from continental margins[2].

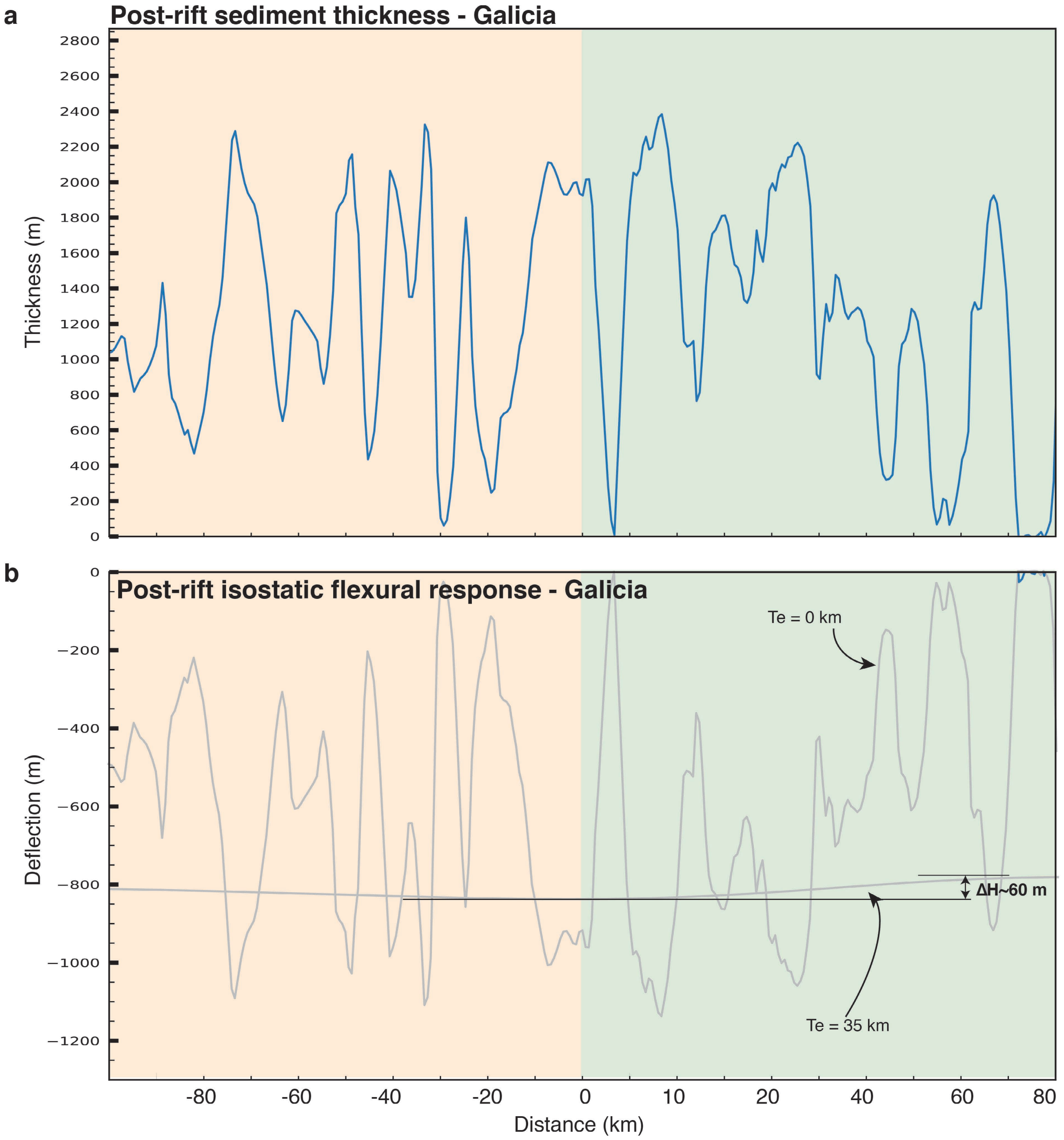

Fig. S3.

Isostatic flexural response to post-rift sediment loading along the Galicia transitional exhumed mantle domain. a, Post-rift sediment thickness; **b,** Post-rift isostatic flexural response. Because the proximity with the continental lithosphere we select 35 km thick elastic thickness that slightly overestimates the result of the following relationship $Te = (2.7 \pm 0.15)\sqrt{\Delta t}$ (Δt in Ma, here 121 Ma = age of crustal breakup) that is defined for oceanic lithosphere far from continental margins[2].

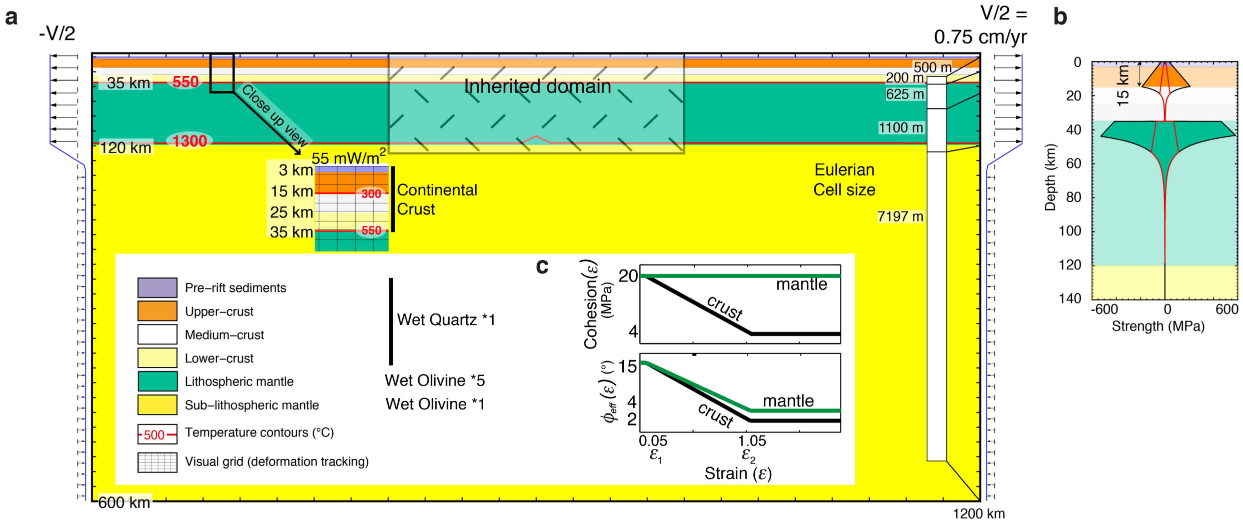

Fig. S4.

Model setup. a, initial rheological layered structure, position and size of the inherited domain, kinematic boundary conditions, initial thermal state. **b**, the strength profile for a strain rate of $2.6 \cdot 10^{-16}$ s^{-1}. c, frictional-plastic strain softening. Mechanical and thermal parameters are listed in table S1 and boundary conditions in table S2.

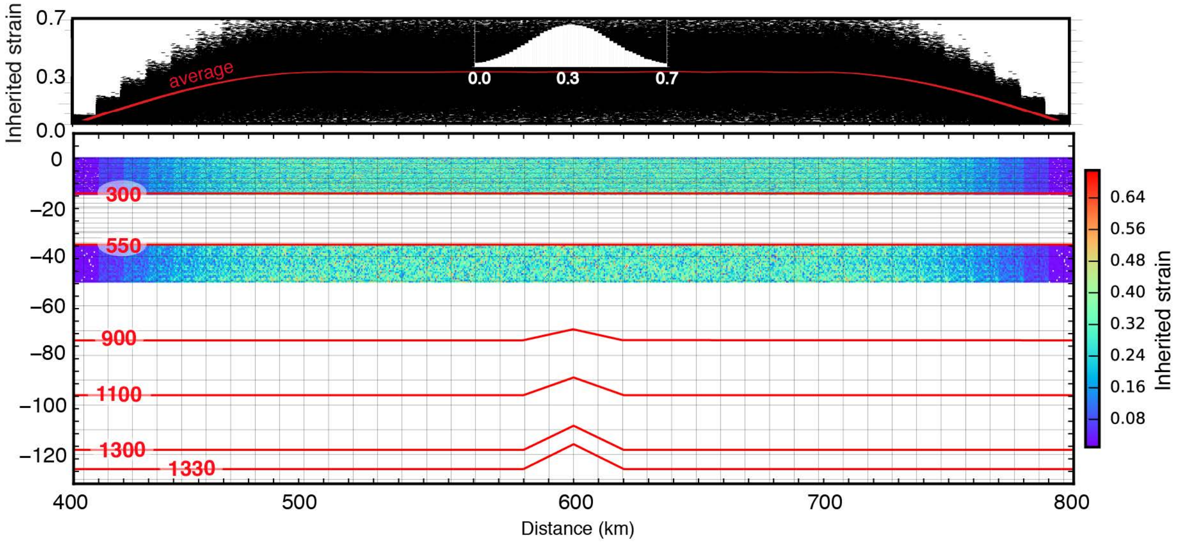

Fig. S5.

Inherited strain distribution carried by Lagrangian particles at the start of the model. Inherited domain is centered between 400 km and 800 km. Bottom: distribution of the initial accumulated strain stored on each particle. Top: statistical distribution of the initial accumulated strain in space (black dots) and globally (white histogram). The accumulated strain translates into weakened frictional properties through the strain dependence of friction and cohesion (Fig. S4 and table S1). This inherited weak domain allows more freedom for faults to localize deformation in comparison to a single weak seed. A small thermal anomaly along the lithosphere-asthenosphere boundary is defined in the center in order to control the position of initial viscous necking.

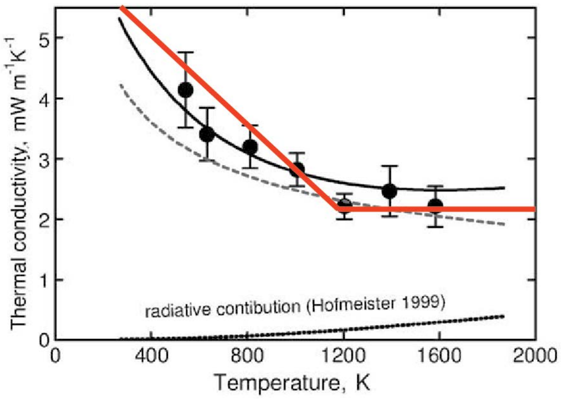

Fig. S6.

Variation of thermal conductivity with temperature for forsterite. The data points and their uncertainties follow Schatz and Simmons (1972)[3]. The solid line is calculated based on McKenzie et al.[4] and the dashed line from the expression of Xu et al. (2004)[5]. The lower values of conductivity of Xu et al. (2004)[5] based on multianvil contact measurements may result from contact losses and radiative gains in comparison with laser-flash measurements[6] that have a higher accuracy.

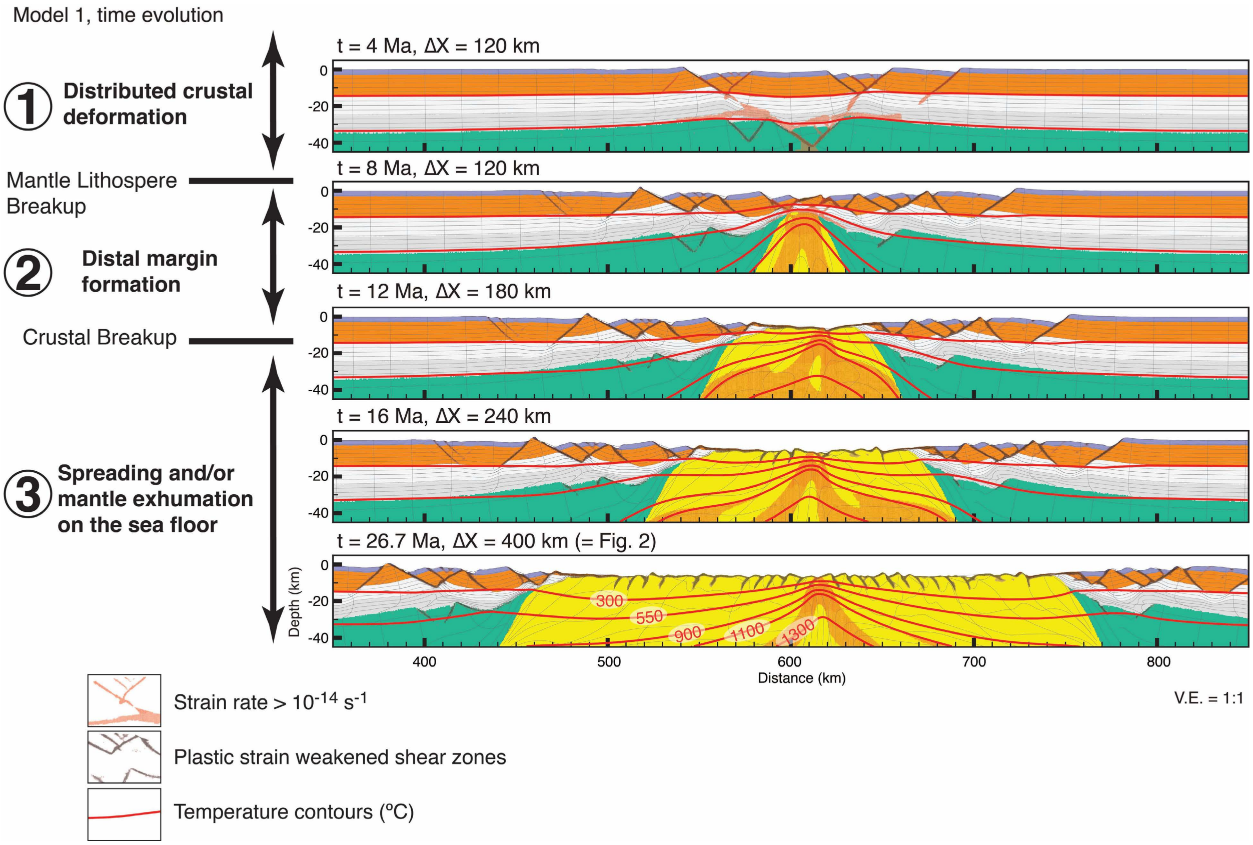

Fig. S7.

Time evolution of reference Model M1 presented in the main text (Fig. 2 and Fig. 4). This model is characterized by wet quartz crustal rheology and wet olivine sub-lithospheric mantle rheology. No surface processes. See tables S1 and S2 for details on model parameters.

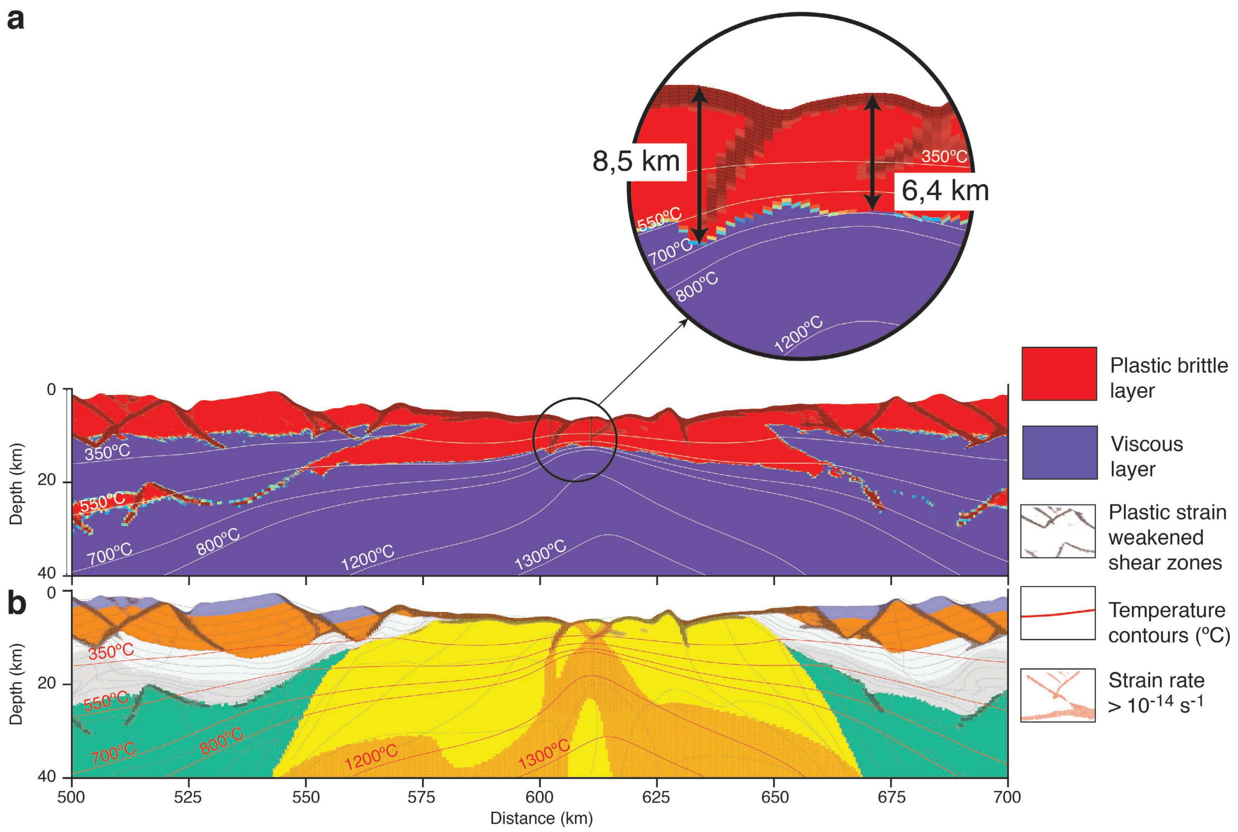

Fig. S8.

Brittle thickness in the exhumed mantle domain of model M1 (15 mm/yr full extension rate). a, domains of brittle and viscous mechanical behavior soon after crustal breakup. **b,** material colors showing the domain of active mantle exhumation at the seafloor. Shown are upper crust (orange), middle crust (white), lower crust (light yellow), pre-rift sediments (purple), lithospheric mantle (green), sub-lithospheric mantle (yellow), overlay of weakened frictional-plastic shear zones (grey).

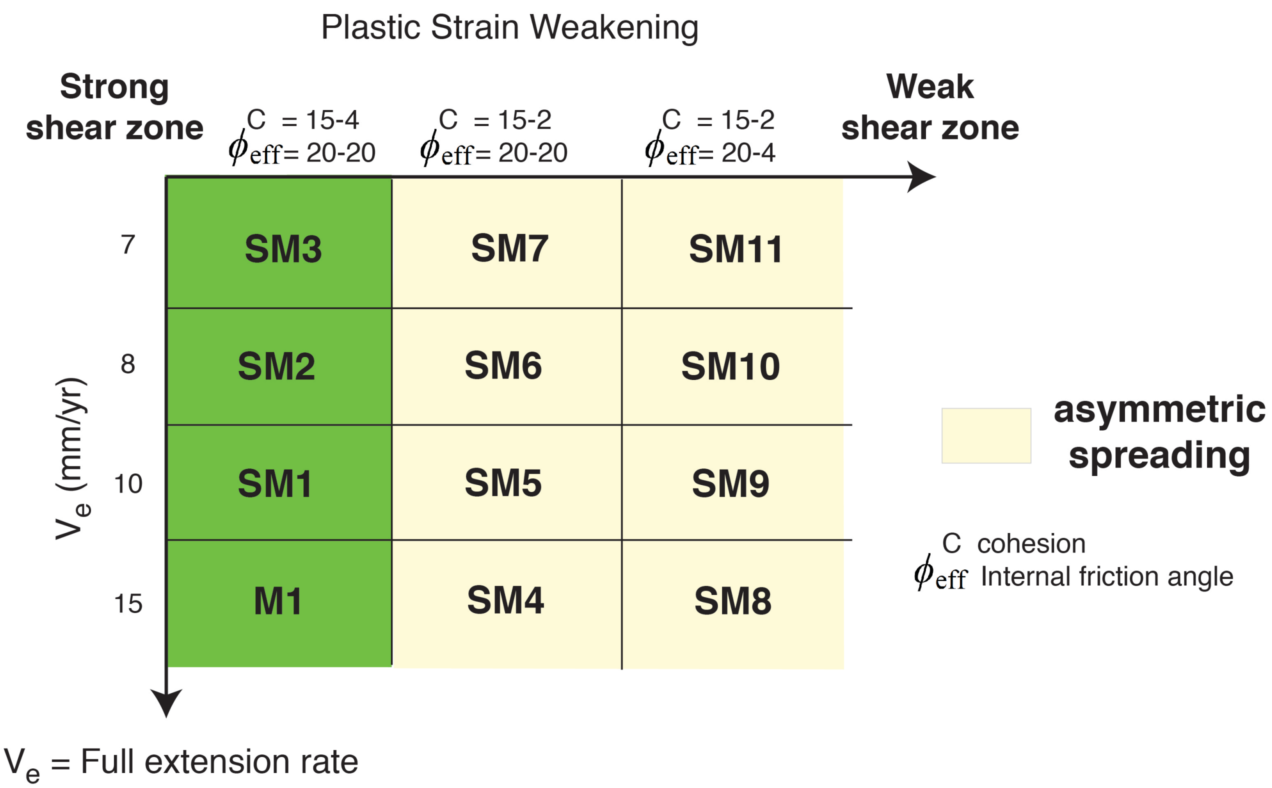

Fig. S9.
Set of models exploring the sensitivity of model behavior to spreading rate versus fault strength. Fault strength is described in terms of plastic strain weakening parameters by increasing the final cohesion and/or final friction angle of mantle material after strain weakening. **M1, SM1-3**: strong mantle shear zones. Green column represents models with symmetric spreading (Fig. S10). **SM4-7**: Intermediate strength mantle shear zones (Fig. S11). **SM8-11**: Weak mantle shear zones (Fig. S12).

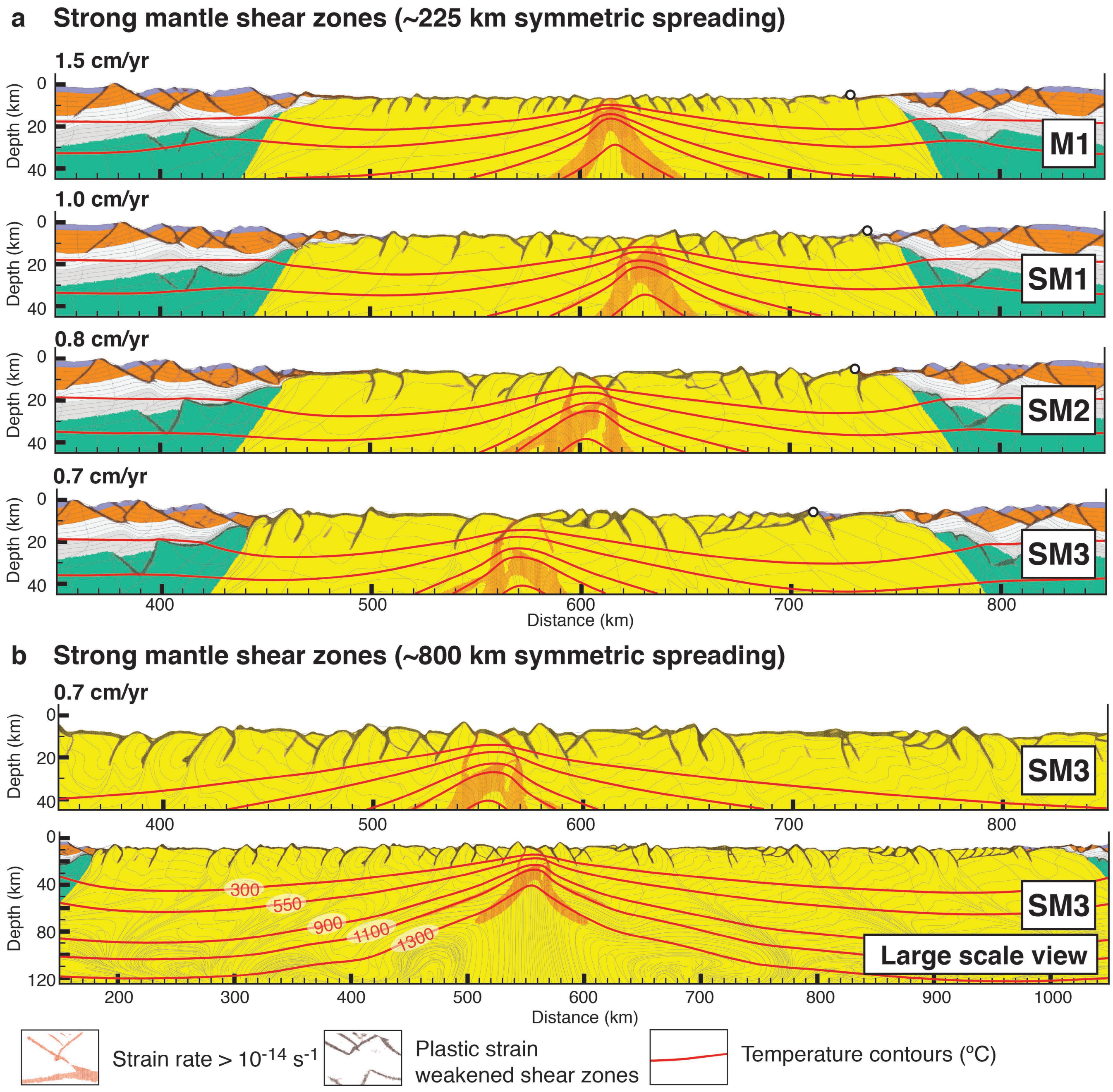

Fig. S10.

Sensitivity of model behavior to spreading rate – Strong mantle shear zones case. Snapshots of material colors with (**a**) about 225 km symmetric spreading and (**b**) about 800 km symmetric spreading for the slowest spreading rate 7 mm/yr shown here. The reference model M1 (main text) has the highest spreading rate 15 mm/yr. Shown are upper crust (orange), middle crust (white), lower crust (light yellow), pre-rift sediments (purple), lithospheric mantle (green), sub-lithospheric mantle (yellow), overlay of weakened frictional-plastic shear zones (grey).

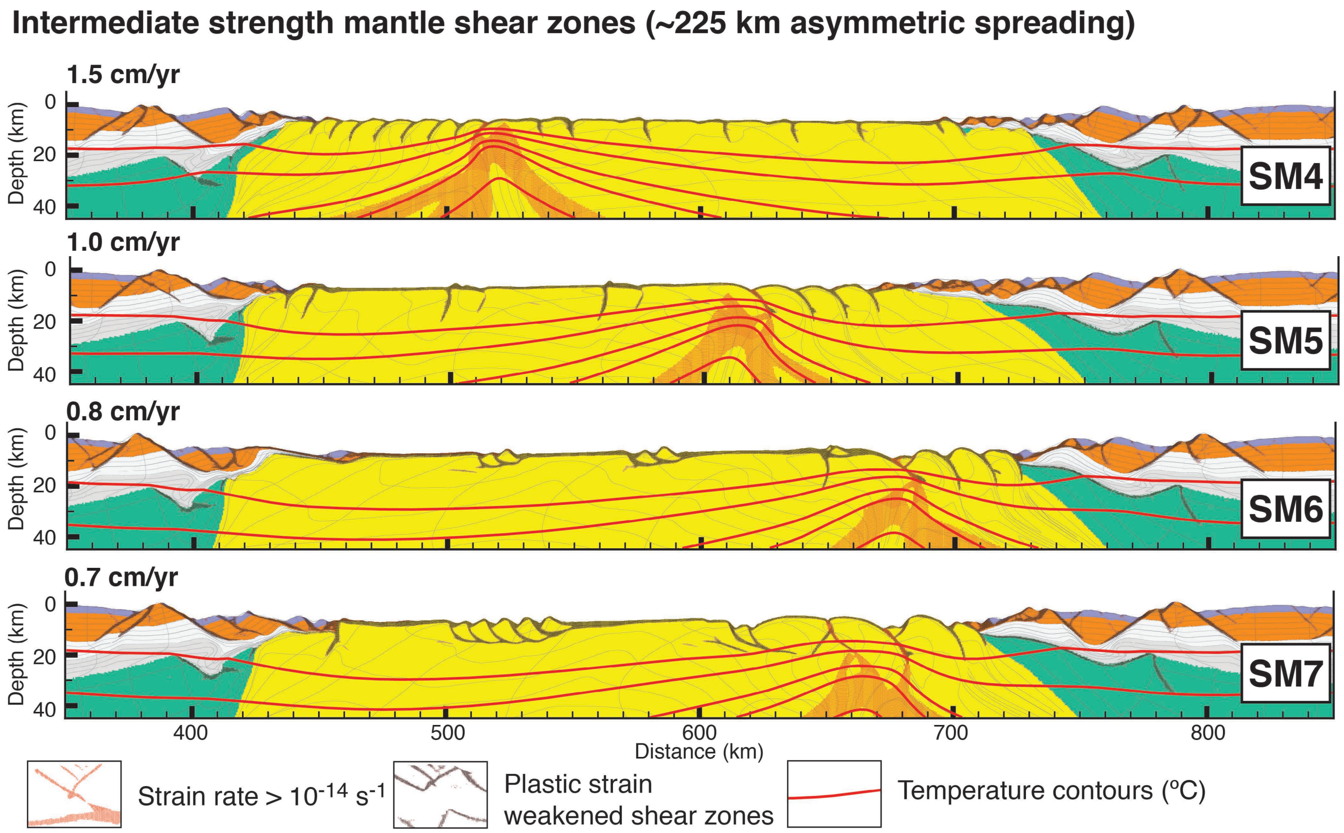

Fig. S11.

Sensitivity of model behavior to spreading rate – Intermediate strength mantle shear zones case. Snapshots of material colors with about 225 km asymmetric spreading. Contours of isotherms in degrees Celsius (300ºC, 550ºC, 900ºC, 1100ºC, 1300ºC). Shown are upper crust (orange), middle crust (white), lower crust (light yellow), pre-rift sediments (purple), lithospheric mantle (green), sub-lithospheric mantle (yellow), overlay of weakened frictional-plastic shear zones (grey).

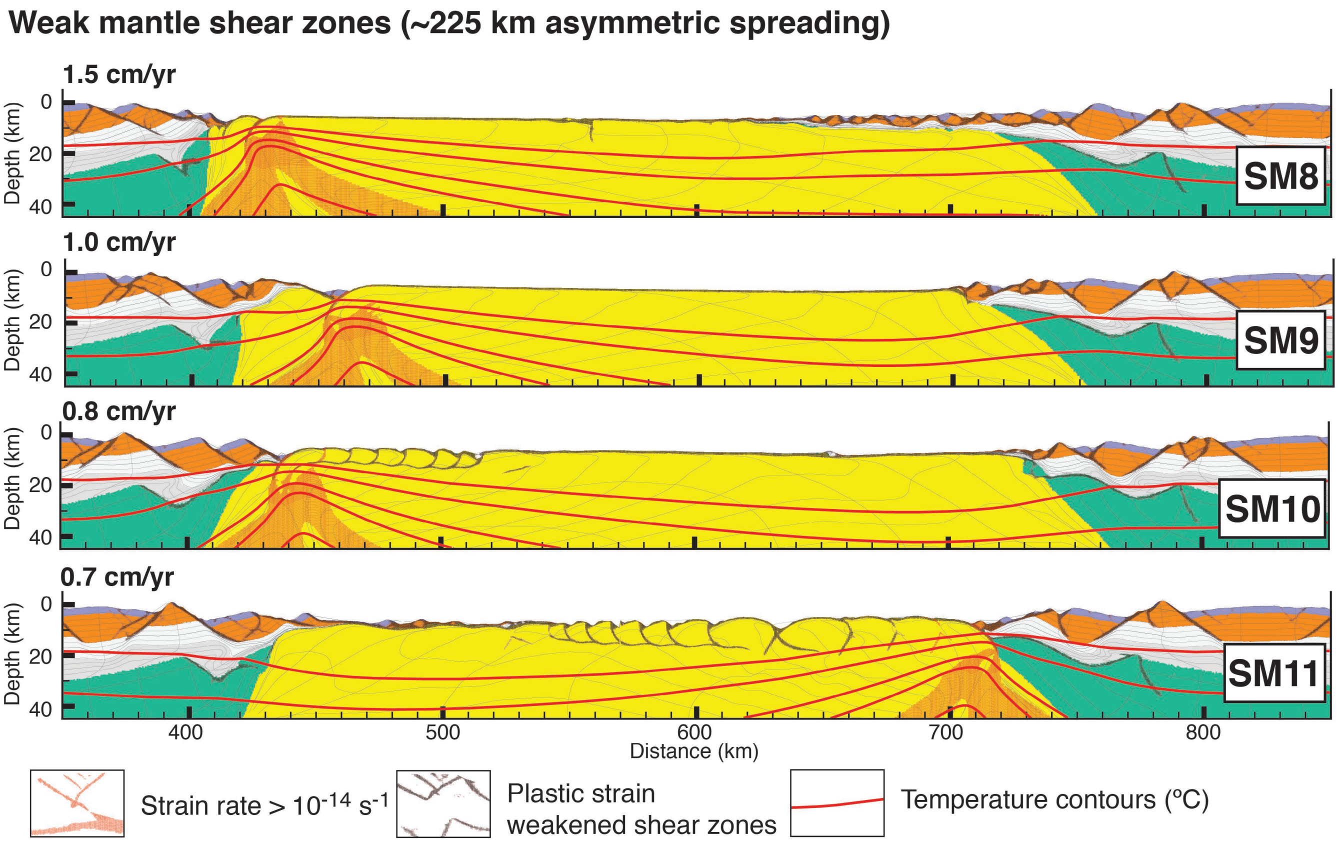

Fig. S12.

Sensitivity of model behavior to spreading rate – Weak mantle shear zones case. Snapshots of material colors with about 225 km asymmetric spreading. Contours of isotherms in degrees Celsius (300ºC, 550ºC, 900ºC, 1100ºC, 1300ºC). Shown are upper crust (orange), middle crust (white), lower crust (light yellow), pre-rift sediments (purple), lithospheric mantle (green), sub-lithospheric mantle (yellow), overlay of weakened frictional-plastic shear zones (grey).

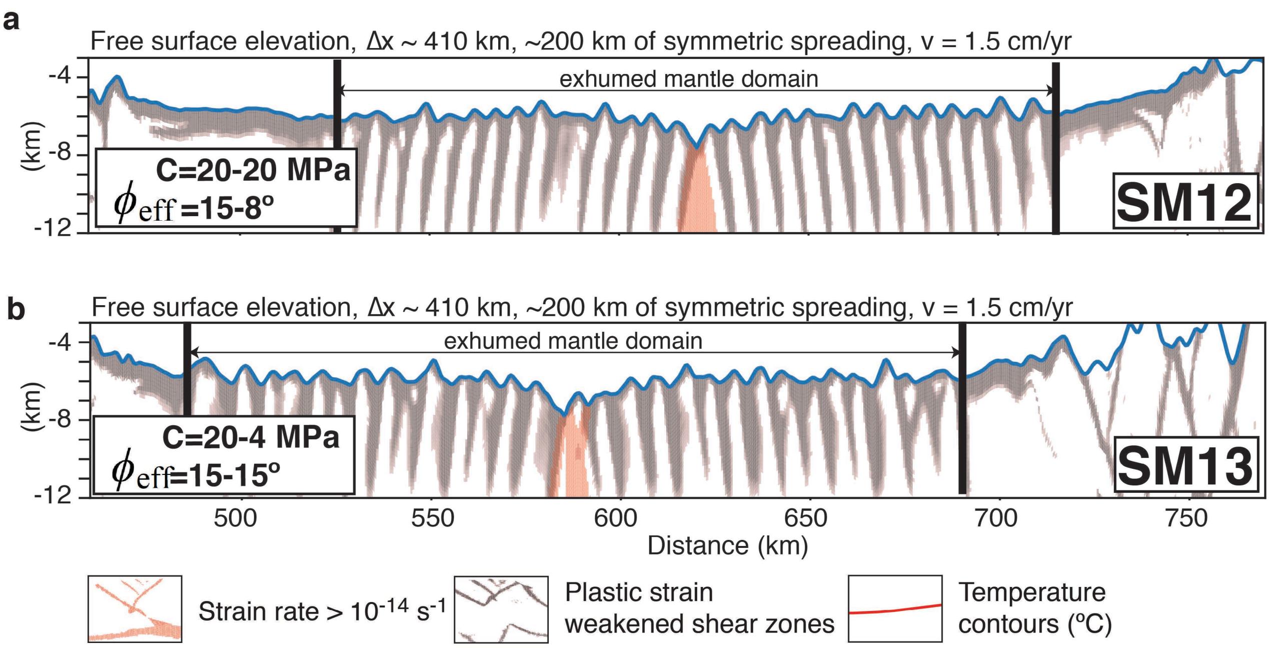

Fig. S13.

Model behavior with reduced strain weakening in the mantle. Free surface elevation and strain weakened shear zones are plotted. Vertical bold lines represent the most distal rifted continental crust material. The models presented here only differ from the reference model M1 (main text) in the fact that the mantle is characterized by a reduced strain weakening. The reduced strain weakening in mantle enhances lower wavelength and smaller amplitudes owing to stronger shear zones and smaller fault offset during mantle exhumation. **a,** amplitude is 0.5±0.3 km and wavelength is 6.8±1.7 km on average. **b,** amplitude is 0.6±0.4 km and wavelength is 7.1±1.6 km on average.

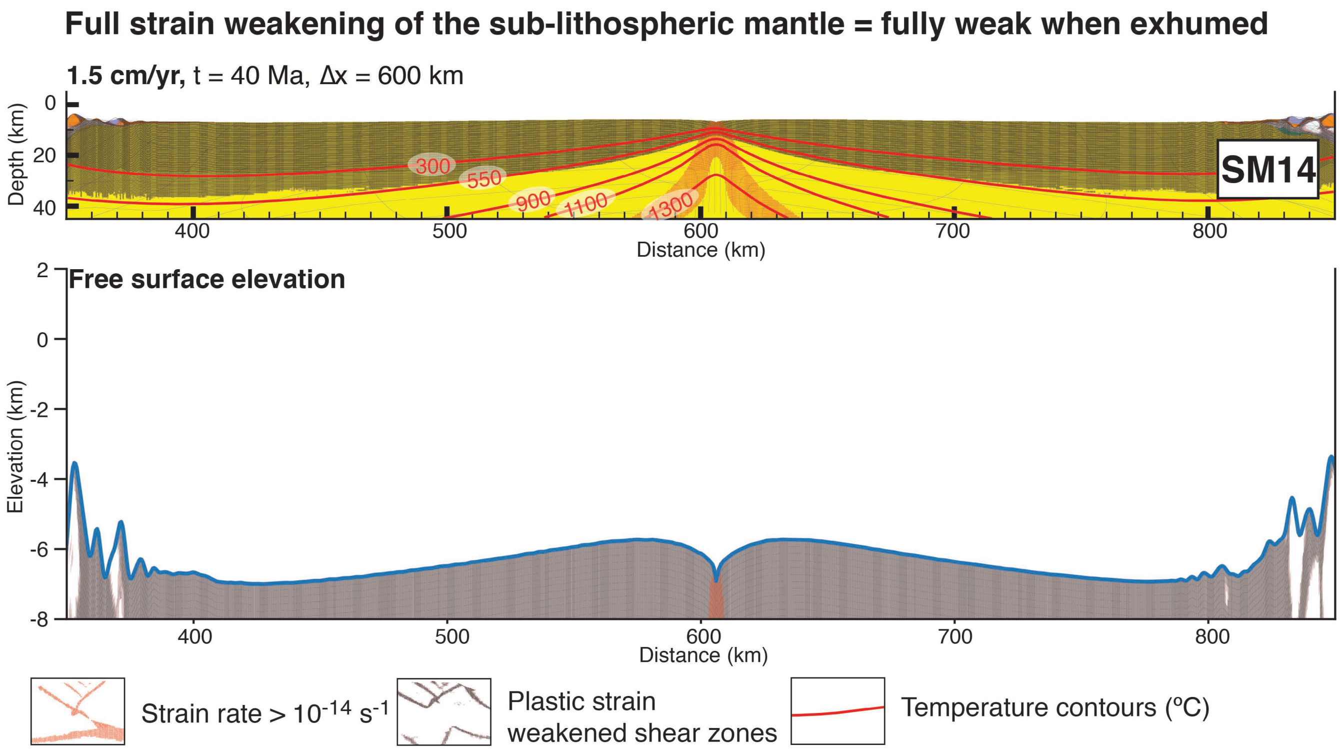

Fig. S14.

Model behavior with the absence of strain weakening in the brittle layer of the exhumed mantle. The model presented here only differs from the reference model M1 (main text) in the fact that the mantle is fully weakened when reaching the shallowest brittle layer (cohesion is constant 20 MPa; friction angle=15º to 4º after strain weakening). The absence of strain weakening in the brittle layer disables the strain localization resulting in a smooth topography owing to the absence of fault-related topography. **On top:** snapshots of material colors with about 400 km symmetric spreading. Shown are upper crust (orange), middle crust (white), lower crust (light yellow), pre-rift sediments (purple), lithospheric mantle (green), sub-lithospheric mantle (yellow), overlay of weakened frictional-plastic shear zones (grey). **Bottom:** free surface elevation and strain weakened shear zones.

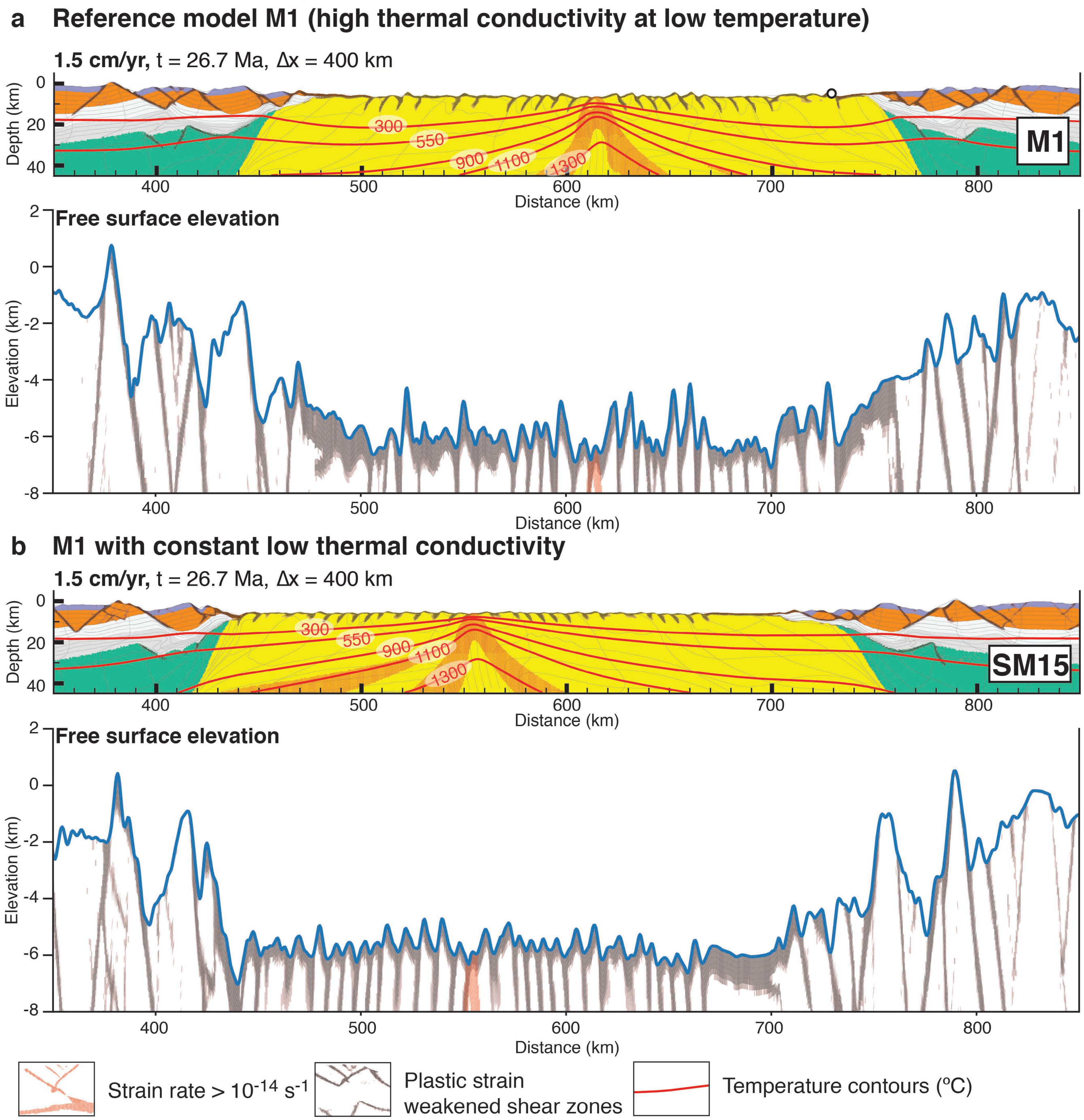

Fig. S15.

Sensitivity of model behavior to thermal conductivity. (a) The reference model M1 (main text) includes temperature dependent thermal conductivity in the mantle, i.e. higher thermal conductivity at low temperature (Fig. S6). **(b)** The second model exhibits a constant thermal conductivity of 2.25 W/m/K in all material. The amplitude of mantle highs, the wavelength of fault shear zones and fault offset decrease in the second case owing to slower cooling and thinner brittle thickness. **On top:** snapshots of material colors with about 225 km symmetric spreading. Shown are upper crust (orange), middle crust (white), lower crust (light yellow), pre-rift sediments (purple), lithospheric mantle (green),

sub-lithospheric mantle (yellow), overlay of weakened frictional-plastic shear zones (grey). **Bottom:** free surface elevation and strain weakened shear zones.

Parameter	Symbol	Units	Upper crust (0–25 km)	Lower crust (25–35 km)	Lithospheric mantle	Sub-lithospheric mantle
**Rheological parameters**						
Effective viscosity range	μ_{eff}	Pa·s	10^{18}–10^{27}	10^{18}–10^{27}	10^{18}–10^{27}	10^{18}–10^{27}
Angle of internal friction (Initial-Final)	ϕ_{eff}	—	15-2°		15-4°	
Cohesion (Initial-Final)	C	MPa	20-4		20-20	
Strain weakening range	—	—	0.05–1.05			
Flow law	—	—	WQtz[a]		WO[b]	
Scaling factor	f	—	1		5	1
Power law exponent	n	—	4		3	
Activation energy	Q	$kJ·mol^{-1}$	222.81		429.83	
Constant	A	$Pa^{-n}·s^{-1}$	$8.574×10^{-28}$		$1.758×10^{-14}$	
Activation volume	V	$m^3·mol^{-1}$	0.0		$15×10^{-6}$	
Density at T_0= 273 K	ρ_0	$kg·m^{-3}$	2750	2900	3300	
**Thermal parameters**						
Thermal conductivity	k	$W·m^{-1}·K^{-1}$	2.25	2.25	2.25-5.6[d]<br>2.25–51.46	
Heat capacity	c_p	$J·K^{-1}·kg^{-1}$	818.18	775.86	681.81	
Thermal diffusivity[c]	κ	$m^2·s^{-1}$	$1.0·10^{-6}$		1 - $2.5×0^{-6}$ [d]<br>1 - $22.87×10^{-6}$	
Heat production rate	H	$W·m^{-3}$	$1.12×10^{-6}$	$0.48×10^{-6}$	0	
Thermal expansion	α_T	K^{-1}	$3.1×10^{-5}$			

Table S1.

Rheological and thermal properties. [a, b] Flow laws are based on power law with creep parameters from wet quartz (WQtz)[7] and wet olivine (WO)[8]. [c] $\kappa=k/\rho c_p$. [d] from 876ºC to 0ºC (Fig. S4).

Boundary conditions			
Surface temperature	T_{surf}	°C	0
Pre-rift sediments thickness	$h_{pre\text{-}sed}$	km	3
Initial Moho depth	d_{moho}	km	35
Moho temperature	T_{moho}	°C	550
LAB depth	d_{LAB}	km	120
LAB temperature	T_{LAB}	°C	1,328
Basal temperature	T_{LM}	°C	1,520
Extension velocity	V_{ext}	cm/yr	1.5

Table S2.
Boundary conditions. LAB = Lithosphere-Asthenosphere Boundary.

Method	Mineral	Age (Ma)	Closing temperature	Ref.
U/Pb on zircons	Chlorite schist	122.1±0.3	About 600ºC	Schärer et al., 1995[9]
U/Pb on zircons	Chlorite schist	121.7±0.4		Schärer et al., 2000[10]
Ar/Ar	Post-kinematic brown amphibole in a diorite dyke	122.0±0.6		Féraud et al., 1988[11]
Ar/Ar	Syn-tectonic plagioclase neoblasts	117.7±0.9	About 200ºC	Boillot et al., 1989[12]

Table S3.
Dating of mantle rocks on top of the peridotite ridge at IODP site 637.

Additional Movies

All movies of the models exploring the sensitivity to strain weakening, full extension rate and thermal conductivity (Fig. S9 to S15) are accessible through FigShare[13].

Supplementary references

1. Winterbourne, J., Crosby, A., White, N. Depth, age and dynamic topography of oceanic lithosphere beneath heavily sedimented Atlantic margins. *Earth and Planetary Science Letters* **287**, 137-151 (2009).

2. Calmant, S., Francheteau, J., Cazenave, A. Elastic Layer Thickening with Age of the Oceanic Lithosphere - a Tool for Prediction of the Age of Volcanos or Oceanic-Crust. *Geophysical Journal International* **100**, 59-67 (1990).

3. Schatz, J. F., Simmons, G. Thermal-Conductivity of Earth Materials at High-Temperatures. *Journal of Geophysical Research* **77**, 6966-6983 (1972).

4. McKenzie, D., Jackson, J., Priestley, K. Thermal structure of oceanic and continental lithosphere. *Earth and Planetary Science Letters* **233**, 337-349 (2005).

5. Xu, Y. S., Shankland, T. J., Linhardt, S., Rubie, D. C., Langenhorst, F., Klasinski, K. Thermal diffusivity and conductivity of olivine, wadsleyite and ringwoodite to 20 GPa and 1373 K. *Physics of the Earth and Planetary Interiors* **143**, 321-336 (2004).

6. Hofmeister, A., Branlund, J. Thermal conductivity of the Earth. In: *Treatise on Geophysics* (ed Elsevier). 2nd edn (2015).

7. Gleason, G. C., Tullis, J. A Flow Law for Dislocation Creep of Quartz Aggregates Determined with the Molten-Salt Cell. *Tectonophysics* **247**, 1-23 (1995).

8. Karato, S., Wu, P. Rheology of the Upper Mantle - a Synthesis. *Science* **260**, 771-778 (1993).

9. Scharer, U., Kornprobst, J., Beslier, M. O., Boillot, G., Girardeau, J. Gabbro and Related Rock Emplacement beneath Rifting Continental-Crust - U-Pb Geochronological and Geochemical Constraints for the Galicia Passive Margin (Spain). *Earth and Planetary Science Letters* **130**, 187-200 (1995).

10. Scharer, U., Girardeau, J., Cornen, G., Boillot, G. 138-121 Ma asthenospheric magmatism prior to continental break-up in the North Atlantic and geodynamic implications. *Earth and Planetary Science Letters* **181**, 555-572 (2000).

11. Feraud, G., Girardeau, J., Beslier, M. O., Boillot, G. Ar-39-Ar-40 Dating of the Emplacement of the Peridotites Bounding the Galicia Margin (Spain). *Cr Acad Sci Ii* **307**, 49-55 (1988).

12. Boillot, G., Feraud, G., Recq, M., Girardeau, J. Undercrusting by Serpentinite beneath Rifted Margins. *Nature* **341**, 523-525 (1989).

13. Theunissen, T. and Huismans, R. S. Mantle exhumation at magma-poor rifted margins controlled by frictional shear zones – Model animations. *figshare* http://dx.doi.org/10.6084/m9.figshare.17153264 (2022).
