# Supplementary material for: Mantle exhumation at magma-poor rifted margins controlled by frictional shear zones
Source: Nat Commun. 2022 Mar 28;13:1634. doi: 10.1038/s41467-022-29058-1 (PMC8960832; doi:10.1038/s41467-022-29058-1)
Supplement: Supplementary file 3 — Description of Additional Supplementary Files [file 41467_2022_29058_MOESM3_ESM.pdf]

Description of Additional Supplementary Files

File name: Supplementary Movie 1 (MovieS1_M1_PTt_path.mp4)
Description: Large scale animation of the reference model M1 presented in the main text. Shown are upper crust (orange), middle crust (white), lower crust (light yellow), pre-rift upper-crust layer (purple), lithospheric mantle (green), sub-lithospheric mantle (yellow), overlay of weakened frictional-plastic shear zones (grey), overlay of strain rate higher than 10-14 s-1, contours of isotherms 350ºC, 550ºC, 800ºC, 1200ºC and 1300ºC. The Pressure-Temperature-time (PTt) history of the first peridotite ridge associated with crustal breakup in our model (Fig. 3A, ridge 1) is shown on the right side of the animation. The tracked point is drawn with a black contoured white point in the animation.

File name: Supplementary Movie 2 (MovieS2_M1_zoom.mp4)
Description: Upper-crustal scale animation of the reference model M1 presented in the main text. Shown are upper crust (orange), middle crust (white), lower crust (light yellow), pre-rift upper-crust layer (purple), lithospheric mantle (green), sub-lithospheric mantle (yellow), overlay of weakened frictional-plastic shear zones (grey), overlay of strain rate higher than 10-14 s-1, contours of isotherms 350ºC and 550ºC.
